# Paclitaxel-induced cognitive impairment: mechanistic insights

**DOI:** 10.3389/fphar.2025.1684006

**Published:** 2025-09-22

**Authors:** Ahmad Hamad Alhowail

**Affiliations:** Department of Pharmacology and Toxicology, College of Pharmacy, Qassim University, Buraydah, Saudi Arabia

**Keywords:** paclitaxel, chemotherapy, neurobehavior, inflammation, oxidative stress, synaptic plasticity

## Abstract

Paclitaxel, a cornerstone taxane for solid-tumor chemotherapy, frequently precipitates long-lasting cognitive deficits, which are collectively termed chemotherapy-related cognitive impairment and often referred to as chemobrain or chemofog. Although the precise mechanisms are still being fully elucidated, a growing body of evidence from preclinical and clinical studies points to several key pathways and cellular targets. Evidence suggests that paclitaxel exerts potential neurotoxic effects through various mechanisms of action that stabilize microtubules and reduce their dynamicity, promoting mitotic halt and cell death. In this review, the major mechanisms and effects of paclitaxel are concisely summarized; these include neuroinflammation, oxidative stress, impaired neurogenesis and synaptic plasticity, neuronal apoptosis, hormonal imbalance, calcium dysregulation, altered white matter integrity, and mitochondrial dysfunction. By synthesizing published mechanistic data, this review highlights emerging molecular targets and experimental therapeutics to help prevent and mitigate paclitaxel-induced cognitive impairment.

## Graphical Abstract

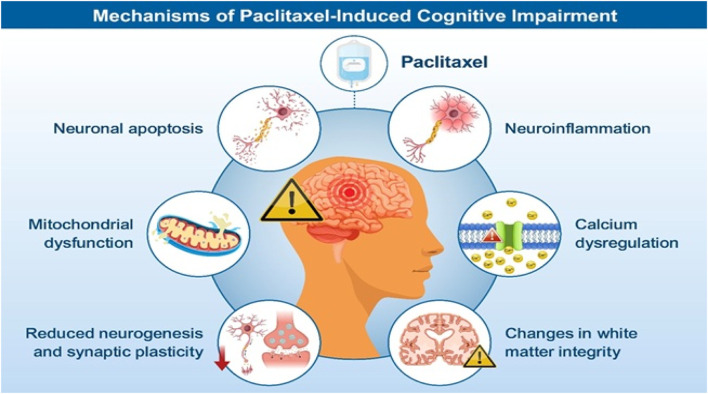

## 1 Introduction

Paclitaxel is a highly effective chemotherapeutic agent widely used in the treatment of various cancers, including breast, ovarian, lung, head, and neck cancers (Abu Samaan et al., 2019). The primary mechanism of action of paclitaxel involves microtubule stabilization, which disrupts cell division and induces apoptosis in rapidly proliferating cancer cells (Kaveh Zenjanab et al., 2024). However, chemotherapy-induced cognitive impairment or chemobrain is a significant and debilitating side effect in many patients with cancer (Murillo et al., 2023). Paclitaxel-induced cognitive impairment can manifest as deficits in memory, attention, executive function, processing speed, and visual–spatial abilities, severely affecting quality of life in patients (Tang et al., 2022). Although the limited ability of paclitaxel to cross the intact blood–brain barrier initially suggested indirect mechanisms of action, research now indicates that paclitaxel can indeed reach brain tissue, particularly the hippocampus, at concentrations sufficient to induce neurotoxicity (Lee et al., 2023). Multiple, often interconnected, mechanisms likely contribute to paclitaxel-induced cognitive impairment (Tang et al., 2022). For example, paclitaxel can alter the integrity of the blood–brain barrier, potentially allowing harmful substances to enter the brain and contributing to neurotoxicity (Patai et al., 2025).

In this review, previous data on the mechanisms of toxicity associated with paclitaxel-induced cognitive impairment are synthesized to enhance our understanding of its toxic effects on bodily systems and organs, with the goal of improving strategies for managing the resulting dysfunction. Understanding the mechanisms underlying paclitaxel-induced cognitive impairment is crucial for developing effective preventive and therapeutic strategies.

This review involved a comprehensive electronic search of the MEDLINE database, Scopus, Web of Science, and Google Scholar, targeting literature including experimental studies, randomized controlled trials, observational studies, and case reports from 2006 to 2025, with a focus on both human and animal studies related to paclitaxel and cognitive impairment. The search parameters were as follows: (paclitaxel, animal, human OR behavior, animal/physiology OR paclitaxel induce cognitive impairment/dysfunction). The search results were meticulously filtered by examining the titles and abstracts to ensure the inclusion of studies specifically involving rats, mice, and human subjects.

## 2 Neurobehavioral impairment

Cognitive function is assessed through learning and memory evaluations. Different behavioral tasks are associated with specific brain regions, reflecting the functionality of each region (Sridhar et al., 2023). Therefore, alterations in neurons within a particular brain region can affect specific behavioral functions; for example, adverse effects on the hippocampus can impact learning and memory retrieval (Albadawi, 2025; Li et al., 2023). In addition, overactivation of the amygdala can lead to anxiety and insomnia, which also affect memory function (Song, 2023). Clinical evidence of the effect of paclitaxel on cognitive dysfunction is limited (Smith et al., 2017). However, the effects of paclitaxel have been investigated in several rodent models of chemobrain (Li et al., 2018; Smith et al., 2017). For example, paclitaxel significantly reduced memory and increased depression-like behavior in mice, as measured by novel object recognition and sucrose preference at two time points (Smith et al., 2017); these effects were independent of illness. Furthermore, recent studies have shown that paclitaxel can reduce memory in mice in the Morris water maze, object recognition task, and open-field exploration, although not in the elevated plus maze (Huehnchen et al., 2017; Li et al., 2018; Nguyen et al., 2021; Walker et al., 2017). Paclitaxel also impaired reversal learning tasks (Smith et al., 2017), with rats treated with paclitaxel unable to rapidly adapt to new experimental contingencies. In contrast, paclitaxel did not impair spatial or episodic memory, with paclitaxel-treated rats no more susceptible to cognitive challenges than control rats (Smith et al., 2017).

## 3 Neuroinflammation

Inflammation is an immune response to infection, injury, and other harmful stimuli. In the brain, this process is predominantly mediated by microglia and astrocytes (Kölliker-Frers et al., 2021) and plays a crucial role in brain function during both health and disease (Biswas, 2023; Kölliker-Frers et al., 2021). Inflammation can be acute or chronic, where acute inflammation is a short, protective response, while chronic inflammation persists long-term, activating immune responses that can damage neurons and disrupt brain function, with a link to neurological disorders (Chen et al., 2017). Elevated pro-inflammatory cytokines, such as IL-6, IL-1β, and TNF-α, can impair cognition by affecting synaptic and neurotransmitter functions, as seen in Alzheimer’s disease, Parkinson’s disease, epilepsy, multiple sclerosis, traumatic brain injury, and post-chemotherapy (Goshi et al., 2025; Zhang et al., 2025). Chronic neuroinflammation, characterized by microglial activation and cytokine release, disrupts synaptic plasticity and leads to neuronal death, ultimately causing memory and learning deficits (Adamu et al., 2024). Previous findings have shown that chemotherapy-related increases in cytokines, especially IL-1β, TNF-α, and IL-4, are strongly linked to cognitive decline in patients with breast cancer, showing a connection between systemic inflammation and cognitive impairment after chemotherapy (Wu et al., 2025). In addition, microglia, the resident immune cells of the central nervous system (CNS), play a critical role in neuroinflammation (Qin et al., 2023).

Paclitaxel-induced neuroinflammation is a key mechanism contributing to cognitive impairment in patients with cancer undergoing chemotherapy (Liu et al., 2024). Paclitaxel activates microglia and astrocytes in the CNS, leading to the release of pro-inflammatory cytokines such as TNF-α, IL-1β, and IL-6 (Klein et al., 2022). This neuroinflammatory response disrupts normal neuronal function, impairs synaptic plasticity, and leads to neuronal death (DiSabato et al., 2016). Additionally, paclitaxel induces oxidative stress by increasing reactive oxygen species (ROS) production and nitric oxide, and decreasing antioxidant defenses, further exacerbating neuroinflammation (Duggett et al., 2016). The inflammatory cascade triggered by paclitaxel also affects blood–brain barrier integrity, allowing peripheral inflammatory mediators to enter the brain; these cytokines directly impair neuronal function, synaptic plasticity, and neurogenesis (Klein et al., 2022). Sustained neuroinflammation ultimately results in cognitive deficits, particularly in memory, attention, and executive function (Lecca et al., 2022).

## 4 Oxidative stress

Oxidative stress occurs when the body’s production of ROS and free radicals exceeds its ability to neutralize them with antioxidants (Pizzino et al., 2017). These reactive molecules can damage cellular components, such as DNA, proteins, and lipids, leading to cellular dysfunction and death (Chandimali et al., 2025). The brain is particularly vulnerable to oxidative stress owing to its high oxygen consumption, abundant lipid content, and relatively low antioxidant defenses (Lee et al., 2020). Oxidative stress plays a central role in the aging brain and the development of various neurological diseases (Ionescu-Tucker and Cotman, 2021). Oxidative stress is reportedly elevated following chemotherapy, which may damage the brain and accelerate the aging process (Cauli, 2021). Therefore, understanding the mechanisms by which medication increases ROS formation and induces brain injury can facilitate the development of strategies for preventing damage caused by oxidative stress.

Increased ROS production is a common feature of chemotherapy-induced neurotoxicity (Was et al., 2022). Paclitaxel can disrupt mitochondrial function, resulting in the overproduction of ROS such as superoxide anions and hydroxyl radicals, as well as reactive nitrogen species (Di Carlo and Sorrentino, 2024; Jena et al., 2023). Activation of nicotinamide adenine dinucleotide phosphate hydrogen oxidase (NOX) enzymes (e.g., NOX2, NOX4) in the brain has also been implicated in paclitaxel-induced oxidative stress (Konaté et al., 2020). Paclitaxel primarily works by stabilizing the microtubules, which are crucial for cell division (Chavez et al., 2019). However, this disruption is not limited to cancer cells; it also affects healthy cells, including those of the nervous system (Gornstein and Schwarz, 2014; Sharifi-Rad et al., 2021). This interference can lead to altered mitochondrial morphology and function, which are the primary sources of ROS in cells (Zhou et al., 2020). Additionally, paclitaxel can lower the levels of endogenous antioxidants, such as glutathione, and inhibit the activity of antioxidant enzymes, such as superoxide dismutase and glutathione peroxidase (Chidambaram et al., 2024; Jena et al., 2023). This imbalance between pro-oxidants and antioxidants creates a state of oxidative stress (Jomova et al., 2023). The disruption of microtubules also impairs axonal transport, which is vital for the delivery of ATP and other essential molecules to neurons (Berth and Lloyd, 2023; Shin, 2023). This can lead to an undersupply of ATP and further contribute to oxidative stress, ultimately producing the cognitive deficits observed in chemobrain.

## 5 Impaired neurogenesis and synaptic plasticity

The hippocampus is a critical brain region for learning and memory, and particularly vulnerable to paclitaxel-induced toxicity (Murillo et al., 2023). Long-term potentiation (LTP) describes an increase in synapse strength, indicating learning and memory formation, and is defined as a persistent increase in excitatory postsynaptic current following stimulation (Alhowail et al., 2023). The two major neurotransmitters regulating these processes are glutamate and gamma-aminobutyric acid (GABA), which are responsible for memory consolidation in the brain, particularly the hippocampus (Behuet et al., 2019). This synaptic plasticity process involves several activities that alter synaptic proteins, including glutamate receptors (α-amino-3-hydroxyl-5-methyl-4-isoxazole-propionate and N-methyl-D-aspartate (NMDA), postsynaptic density protein 95 (PSD-95), and other kinases such as calmodulin-dependent kinase II (CaMKII) and protein kinase C (PKC), to modify the synaptic structure (Alhowail and Aldubayan, 2023; de León-López et al., 2025; Kaczmarski et al., 2023). Moreover, paclitaxel can reduce the volume of the hippocampus and impair hippocampus-dependent spatial memory (Huehnchen et al., 2017; Panoz-Brown et al., 2017). Paclitaxel can suppress adult hippocampal neurogenesis, a process by which new neurons are generated in the hippocampus (Panoz-Brown et al., 2017). A reduction in the production and survival of new neurons is strongly associated with cognitive decline (Mostafa et al., 2025). Paclitaxel can also disrupt the synaptic transmission required for synapse strengthening or weakening over time, which is fundamental to learning and memory (Liu et al., 2023). This includes decreased length and thickness of postsynaptic density, reduced dendritic spine density, and decreased density of PSD-95 and brain-derived neurotrophic factor, both of which are crucial for synaptic structure and function (Tang et al., 2022). Alterations in dendritic spine density are also essential for synaptic connections (Tang et al., 2022).

Few studies have investigated the effect of chemotherapy on synaptic plasticity. Synaptic plasticity following paclitaxel treatment has predominantly been evaluated using electrophysiological assessments of miniature excitatory postsynaptic potential. The results showed that LTP was not altered by paclitaxel treatment, although the LTP response was higher than that in the controls. Nashawi et al. (2016) evaluated the effect of paclitaxel treatment on brain slices using a low concentration of paclitaxel, which is similar to the concentration that reaches the brain under *in vivo* conditions, and showed that paclitaxel produced a significantly higher maximal response in LTP. Thus, a potential mechanism underlying paclitaxel-induced chemobrain is the overactivation of intrasynaptic functions (Nashawi et al., 2016).

Similarly, paclitaxel affects synaptic plasticity by increasing presynaptic NMDA receptor (NMDAR) activity, especially that of GluN2A-containing subunits, in the spinal cord, thereby mediating neuropathic pain (Xu et al., 2022). In addition, paclitaxel increases the expression of group I metabotropic glutamate receptors, which play a role in synaptic plasticity and NMDAR regulation (Xie et al., 2017). This activation enhances glutamate release from primary afferent terminals, resulting in increased normal and miniature excitatory postsynaptic currents, and elevates PKC activity, which mediates the phosphorylation of NMDARs (e.g., the GluN1 subunit at Ser896). PKC-dependent phosphorylation enhances NMDAR function, reduces magnesium blocks, and promotes NMDAR trafficking to the plasma membrane, further strengthening synaptic activity (Xie et al., 2016). Moreover, paclitaxel has also been reported to induce neurotoxicity, primarily by affecting neural stem cells and progenitor cells in the hippocampus, which leads to impaired spatial memory (Huehnchen et al., 2017). The suggested mechanism involves an increase in intracellular calcium levels and enhanced activity of calpain and caspase 3/7, suggesting a calcium-dependent mechanism rather than direct effects on synaptic function (Huehnchen et al., 2017). Conversely, paclitaxel treatment did not affect neuronal function or synaptic activity based on LTP measurements in hippocampal preparations from treated mice. This suggests that paclitaxel does not directly impair LTP, and that the observed cognitive decline in paclitaxel-treated mice may be caused by damage in the blood vessels of the brain rather than direct effects on neuronal activation or synaptic plasticity (Ahire et al., 2023).

## 6 Activation of neuronal apoptosis

Apoptosis, also known as programmed cell death, is a tightly regulated physiological process that is essential for maintaining cellular homeostasis by eliminating damaged, dysfunctional, or excess cells without triggering inflammation (Yuan and Ofengeim, 2023). In the CNS, controlled apoptosis is critical during neurodevelopment for synaptic pruning and sculpting of neural circuits (Hollville et al., 2019). However, dysregulation of apoptotic pathways significantly contributes to neurodegenerative diseases and cognitive decline (El Sheikha et al., 2025). Excessive or aberrant neuronal apoptosis leads to the progressive loss of neurons and synapses, particularly in regions involved in memory and cognition, such as the hippocampus and cortex (Hollville et al., 2019). In Alzheimer’s, amyloid-β and hyperphosphorylated tau proteins can activate intrinsic apoptotic pathways, resulting in neuronal death and cognitive impairment (Choi et al., 2023). Thus, understanding the molecular mechanisms governing apoptosis is crucial for developing targeted therapeutic strategies aimed at preventing or slowing cognitive decline associated with aging and neurodegeneration (Mustafa et al., 2024).

Although paclitaxel is known to induce apoptosis, recent research has highlighted the role of necroptosis, a form of programmed necrotic cell death, in chemobrain (Liu et al., 2024). Paclitaxel can activate receptor-interacting protein kinases 1 and 3, and the mixed lineage kinase domain-like pseudokinase signaling pathway, leading to necroptosis in hippocampal neurons (Liu et al., 2024; Zhou et al., 2024). Necroptosis inhibition has protective effects against paclitaxel-induced cognitive deficits in animal models (Liu et al., 2021). In addition, paclitaxel increases the expression of inducible nitric oxide synthase and inflammatory factors, such as TNF-α and IL-β, in hippocampal neurons, which are known markers of cognitive dysfunction (Xu et al., 2022). Experiments in mice have shown that paclitaxel increases necroptosis in hippocampal neurons (Tang et al., 2022).

Paclitaxel can also disrupt intracellular calcium homeostasis by enhancing the interaction between the neuronal calcium sensor-1 protein and inositol-1,4,5-trisphosphate receptor, leading to calcium overload and triggering neuronal cell death pathways, including calpain and caspase 3/7. Lithium, a known modulator of calcium signaling, has shown promise in preventing chemobrain in preclinical models by inhibiting this interaction.

## 7 Hormonal imbalance

Hormones play a vital role in regulating body functions, including the CNS. Alterations in peripheral hormone levels can affect brain function. A reduction in thyroid, insulin, or estrogen levels can affect brain function, including cognitive decline (Kacem et al., 2025; Madhusudhan et al., 2022; Russell et al., 2019). The association between paclitaxel-induced cognitive decline and endocrine system effects has not been fully elucidated. However, a clinical study reported that paclitaxel caused hypothyroidism in several patients, suggesting that paclitaxel can disrupt the hormonal balance (Mie, 2025). Thyroid hormones play essential roles in brain function and development by activating thyroid receptors in the brain (Talhada et al., 2019). Therefore, paclitaxel-induced hypothyroidism may contribute to cognitive deficits. Additionally, paclitaxel may affect the levels of other hormones, such as estrogen, which affect cognition when altered (Miyamoto et al., 2021). Disruption of normal hormonal signaling in the brain by paclitaxel likely interacts with other mechanisms, such as oxidative stress, neuroinflammation, and impaired neuroplasticity, to produce cognitive impairment. However, further research is required to fully characterize the effects of paclitaxel on the endocrine systems that contribute to chemobrain.

## 8 Calcium dysregulation

Calcium plays a crucial role in the nervous system and cognitive functions. Calcium ions (Ca^2+^) function as secondary messengers in neuronal signaling, crucially regulating neurotransmitter release at synaptic terminals; this regulation is fundamental for synaptic plasticity, learning, and memory (Camandola et al., 2012). Additionally, calcium activates enzymes, such as CaMK and PKC, which are vital for memory consolidation and cognitive processes (Brzozowski and Skelding, 2019). Although excessive intracellular calcium can lead to neuronal damage, insufficient calcium levels are associated with cognitive decline (Calvo-Rodriguez et al., 2020). Therefore, maintaining calcium homeostasis is essential for preventing neuronal apoptosis, whereas calcium dysregulation can result in excitotoxicity, contributing to neurodegenerative diseases, such as Alzheimer’s and Parkinson’s disease (Calvo-Rodriguez et al., 2020; Zündorf and Reiser, 2011). As mentioned earlier, paclitaxel can disrupt intracellular calcium homeostasis by enhancing the interaction between the neuronal calcium sensor-1 protein and inositol-1,4,5-trisphosphate receptor, leading to calcium overload in neurons and triggering cell death pathways, including calpain and caspase 3/7. Excessive calcium influx can impair mitochondrial function, increase oxidative stress, and disrupt synaptic plasticity, all of which can contribute to cognitive deficits. Additionally, paclitaxel-induced inflammation and oxidative damage can further exacerbate calcium dysregulation by affecting the calcium channels and pumps (Li et al., 2024). This calcium-mediated neurotoxicity affects the hippocampus with the resulting neuronal death, reduced neurogenesis, and impaired synaptic function in the hippocampus manifesting as memory deficits (Li et al., 2024).

## 9 White matter integrity changes

White matter integrity refers to the structural soundness and organization of the brain’s white matter, which is composed of myelinated nerve fibers that connect various brain regions. These connections are crucial for facilitating efficient communication within the brain. The integrity of the white matter is a fundamental aspect of cognitive functioning. Disruptions in the white matter, whether due to aging, vascular pathology, or neurodegenerative diseases, are strongly linked to cognitive decline (Raghavan et al., 2022). Neuroimaging studies employing magnetic resonance imaging have explored the relationship between white matter structure and cognitive impairment in patients who underwent a chemotherapy regimen comprising uracil, cyclophosphamide, methotrexate, and 5-fluorouracil after surgery, comparing the results with those of patients who did not receive chemotherapy. The results indicated a reduction in both gray and white matter regions after 1 year in the brains of patients who underwent chemotherapy, establishing a clear connection between white matter integrity, cognitive impairment, and chemotherapy (Inagaki et al., 2006).

Moreover, paclitaxel has been linked to detrimental effects on white matter integrity in the CNS. Empirical evidence indicates that paclitaxel administration can result in axonal degeneration, demyelination, and alterations in oligodendrocyte function, collectively leading to disruption of white matter architecture and functionality (Akin et al., 2021; Tasnim et al., 2016). These pathological changes may manifest as cognitive impairment, sensory deficits, and motor dysfunction in patients undergoing paclitaxel-based chemotherapy. Advanced neuroimaging techniques, such as diffusion tensor imaging, have identified reductions in fractional anisotropy and elevations in mean diffusivity within white matter tracts after paclitaxel exposure, indicating compromised structural integrity (Otto et al., 2022). The underlying mechanisms of paclitaxel-induced white matter damage have not yet been reported but may involve mitochondrial dysfunction, oxidative stress, and neuroinflammation (Xu et al., 2022). A comprehensive understanding of these effects is essential for devising strategies to mitigate the neurotoxic consequences of paclitaxel, thereby enhancing quality of life for patients with cancer receiving this treatment.

## 10 Impaired mitochondrial function

Mitochondria, which are far more abundant than energy producers, are central regulators of synaptic plasticity, learning, and memory (Casanova et al., 2023). Their roles in energy supply, calcium buffering, ROS regulation, and dynamic transport are essential for maintaining the functional and structural plasticity of neurons (Walters and Usachev, 2023). Mitochondrial impairment severely compromises the ability of the brain to form memories by disrupting energy supply, calcium balance, and synaptic plasticity (Walters and Usachev, 2023). These impairments not only reduce neuronal efficiency but also contribute to the development of neurodegenerative diseases and age-related cognitive decline (Meng et al., 2025).

Paclitaxel impairs mitochondrial function through multiple mechanisms, leading to cognitive dysfunction. Notably, paclitaxel disrupts mitochondrial morphology and dynamics and interferes with normal energy production (Chavez et al., 2019). This impairment in mitochondrial function enhances ROS generation and oxidative stress, resulting in damage to neuronal proteins, lipids, and DNA. Additionally, by stabilizing microtubules, paclitaxel impairs the axonal transport of mitochondria and other essential molecules along neurons (Shemesh and Spira, 2009). The resulting energy deficits and accumulation of damaged mitochondria contribute to neuronal dysfunction and apoptosis, particularly in metabolically demanding regions such as the hippocampus. Thus, mitochondrial impairment plays a key role in paclitaxel-induced cognitive deficits by disrupting synaptic plasticity, neurogenesis, and overall brain energy metabolism.

While this review offers an overview of the proposed mechanisms underlying paclitaxel-induced cognitive impairment, several limitations warrant consideration. The majority of the existing evidence is derived from preclinical animal models, with limited data available from human studies. Although these models are invaluable for investigating mechanistic pathways, they may not fully replicate the complexity of human neurobiology or the clinical manifestation of cognitive impairment in patients receiving paclitaxel. Additionally, variations in dosing regimens, species-specific responses, and behavioral testing paradigms can influence the translatability of findings. The paucity of well-controlled clinical studies complicates the confirmation of whether the mechanisms identified in animals are directly applicable to humans. Therefore, caution is advised when extrapolating these findings to clinical contexts, and future research involving human participants is necessary to validate and expand the current understanding.

## 11 Discussion

Paclitaxel-induced cognitive impairment is a significant clinical challenge for patients with cancer. The current understanding points to a multifaceted pathogenesis involving neuroinflammation, oxidative stress, impaired neurogenesis and synaptic plasticity, neuronal apoptosis, hormonal imbalance, calcium dysregulation, white matter disruption, and mitochondrial impairment. A comprehensive understanding of these interconnected mechanisms is crucial for developing effective strategies to prevent and treat the debilitating side effects of cancer treatment. This review provides a foundation for future research on emerging molecular targets and experimental therapeutics that can ultimately improve long-term quality of life in patients undergoing paclitaxel-based chemotherapy.
